# Baricitinib and ritlecitinib in alopecia areata: A nationwide prospective real-world registry study

**DOI:** 10.1016/j.jdin.2026.09.010

**Published:** 2026-10-01

**Authors:** Mathias Willaert, Sophie C. van Helmond, Lieke van Vugt, Antoni Gostyński, Tristan H. van Dongen, Sherief R. Janmohamed, Manon Zweers, Tamar E.C. Nijsten, DirkJan Hijnen, Rick Waalboer-Spuij

**Affiliations:** a Department of Dermatology, Erasmus University Medical Center, Rotterdam, The Netherlands; b Department of Dermatology, Maastricht University Medical Center, Maastricht, The Netherlands; c Department of Dermatology, Spaarne Gasthuis, Haarlem, The Netherlands; d Department of Dermatology, Intermedica Kliniek, Wijchen, The Netherlands; e Department of Dermatology, Bernhoven Hospital, Uden, The Netherlands; f Department of Dermatology, Radboud University Medical Center, Nijmegen, The Netherlands

**Keywords:** adults, alopecia areata, baricitinib, epidemiology, JAK inhibitor, real-world data, registry, ritlecitinib

## Abstract

**Background:**

Janus kinase inhibitors baricitinib and ritlecitinib are approved for the treatment of severe alopecia areata (AA). However, prospective real-world data on effectiveness, disease burden, and tolerability are limited.

**Objective:**

To evaluate the real-world effectiveness and tolerability of baricitinib and ritlecitinib in adults with AA.

**Methods:**

In this nationwide, prospective, multicenter registry (Systemic Treatments for Alopecia Areata Registry), adults initiating baricitinib or ritlecitinib between January 2024 and April 2026 were included. The primary outcome was Severity of Alopecia Tool (SALT) score trajectory over 12 months, analyzed using a linear mixed model adjusted for baseline SALT score, AA subtype, age, sex, atopic history, disease duration, prior treatment response, and prior Janus kinase inhibitor use.

**Results:**

A total of 166 patients were included (baricitinib, *n* = 106; ritlecitinib, *n* = 60). SALT scores declined significantly over time in both groups (β = −4.22 points/mo), with no significant difference between treatments. At 12 months, adjusted SALT scores were 33.7 for baricitinib and 33.4 for ritlecitinib, with SALT ≤20 achieved by 30 of 47(64%) and 12 of 24(50%) of patients, respectively. Both treatments were associated with improvements in disease burden and were well tolerated.

**Limitations:**

Nonrandomized design with potential confounding by indication; decreasing sample sizes at later time points reflecting ongoing registry accrual.

**Conclusions:**

Baricitinib and ritlecitinib demonstrated similar scalp hair regrowth in this prospective real-world cohort.

**Capsule Summary**

• Baricitinib and ritlecitinib are approved Janus kinase inhibitors for severe alopecia areata, however, real-world data are limited.
• In this prospective registry of 166 patients, both treatments led to substantial hair regrowth, a favorable safety profile, and improved health-related quality of life, with responses continuing to accumulate beyond 6 months.

## Introduction

Alopecia areata (AA) is a chronic autoimmune disorder causing nonscarring hair loss on the scalp, face, or body. It affects about 2% of people worldwide and often develops early in life.^1^ Hair loss can have a profound impact on self-esteem and psychological well-being.^2^ Many patients report anxiety, depression, and social withdrawal, highlighting the substantial psychosocial burden of the disease.^3^

AA arises from a collapse of hair-follicle immune privilege and a cytotoxic T-cell–mediated attack on anagen follicles, driven by cytokine signaling through the Janus kinase (JAK)–signal transducer and activator of transcription pathway.^4^, ^5^ This insight has led to the development of targeted JAK inhibitors (JAKis), such as baricitinib and ritlecitinib, which have shown robust efficacy in adults with severe AA.^6^, ^7^, ^8^ Continuous therapy supports sustained hair regrowth, suggesting that long-term suppression of immune activity is required to maintain response.^9^ Nevertheless, the management of AA remains a clinical challenge, as treatment responses vary and standardized long-term strategies are still lacking.

Although randomized controlled trials have demonstrated the efficacy and safety of JAKi, their findings reflect highly selected patient populations and limited follow-up periods. Real-world patients often differ in disease duration, comorbidities, and treatment history. Consequently, there is limited real-world evidence on the durability of treatment response and long-term safety of systemic therapy.^10^ Recent retrospective studies^10^, ^11^, ^12^, ^13^, ^14^, ^15^, ^16^ have begun to shed light on these issues but highlight the need for larger, prospective data sets to inform clinical decisions and guideline development.^17^, ^18^

To address this gap, the Systemic Treatments for Alopecia Areata Registry (STA^2^R) was established in the Netherlands. This multicenter, prospective registry collects longitudinal real-world data on adults receiving systemic therapy for AA, the majority of whom are treated with JAKi. The present study reports the first findings from this registry, which was designed to evaluate treatment effectiveness using the Severity of Alopecia Tool (SALT) score as the primary end point, alongside safety and patient-reported quality-of-life outcomes. By prospectively documenting treatment effectiveness, safety, and health-related quality of life (HRQoL) in daily practice, this study aimed to provide valuable insight into real-world management of AA and to support future therapeutic guidelines.

## Methods

### Study design and setting

STA^2^R is a national, multicenter, prospective registry enrolling adults with AA receiving any systemic treatment across dermatology centers in the Netherlands, established in January 2024. Patients were followed according to routine clinical care; data were collected in Castor Electronic Data Capture. Treatment allocation was not randomized; patients and doctors made shared treatment decisions based on clinical profile and personal preference, with baricitinib generally preferred in patients with concomitant moderate-to-severe atopic dermatitis. In the Netherlands, baricitinib received reimbursement in October 2024 and ritlecitinib in December 2024, both requiring a SALT score of ≥50, prior systemic treatment, and an AA episode duration of <8 years. All patients provided written informed consent prior to enrolment.

### Participants

All adults (≥18 years) with a confirmed AA diagnosis enrolled in STA^2^R were eligible. The present analysis includes patients who initiated baricitinib or ritlecitinib between January 2024 and April 2026 with a baseline SALT score >20. Patients who switched between treatments during follow-up were included in the primary analysis for each respective treatments episode.

### Outcome measures

The primary outcome was scalp hair regrowth assessed by the SALT score (0-100) at each follow-up visit. Clinically meaningful response was defined as SALT ≤20, corresponding to at least 80% scalp hair coverage. Secondary outcomes included the change in patient-reported outcome measures over time: disease burden (numeric rating scale [NRS]; 0-10), HRQoL (Dermatology Life Quality Index [DLQI]; 0-30), and disease-specific quality of life (Alopecia Areata Symptom Impact Scale [AASIS]; 0-10). Descriptive outcomes included eyebrow, eyelash, body, and beard hair regrowth (yes/no), and nail involvement (none/partial/complete). Adverse events (AEs) were systematically and actively solicited from patients at each clinical visit and recorded prospectively as part of routine care.

### Statistical analysis

All analyses were performed in R (version 4.5.2). Baseline characteristics were summarized descriptively, stratified by treatment group. Continuous variables are reported as mean (SD) or median (IQR); categorical variables as frequency and percentage. The primary analysis modeled the SALT score over time using a linear mixed model (LMM), selected to account for within-patient correlation across repeated measures and variable follow-up intervals. The model included time since treatment start as a continuous fixed effect, treatment group (baricitinib as reference), and a time × treatment interaction term to test whether trajectories differed between groups. Covariates were selected based on clinical relevance: baseline SALT score, most severe AA subtype ever recorded, age at treatment start, sex, history of atopy, total disease duration, prior non-JAKi response (treatment-naive, prior response, or no prior response), and prior JAKi use. A random intercept per patient was specified. Patients with missing values in any covariate were excluded from the multivariable model. Estimated marginal means were derived at 0, 3, 6, 9, and 12 months. The proportion achieving SALT ≤20 over time was calculated and illustrated using Kaplan-Meier curves. Patients were excluded if treatment data or baseline SALT score were missing. Three sensitivity analyses were performed: (1) inclusion of patients with baseline SALT ≤20; (2) restriction to first treatment episodes only; and (3) complete exclusion of patients who switched between JAKi, including their first treatment episode. Eyebrow, eyelash, body hair, and beard regrowth and nail change resolution were assessed descriptively among patients using a binary classification (present/absent). The proportions of SALT ≤20, NRS, DLQI, and AASIS scores were assigned to fixed time points (0, 3, 6, 9, and 12 months) using a ±1.5-month window and presented as median (IQR) given nonnormal distributions. Each time point was compared with baseline using a Wilcoxon signed-rank test or paired *t* test based on normality of change scores (Shapiro-Wilk, α = 0.05). AEs were summarized descriptively.

## Results

### Patient characteristics

Of 205 patients enrolled in the STA^2^R registry between January 2024 and April 2026, 166 unique patients met the inclusion and exclusion criteria and were included in the primary analysis (baricitinib, *n* = 106; ritlecitinib, *n* = 60; Table I). Baseline characteristics were well balanced between treatment groups. The mean age at treatment start was 37.4 years for baricitinib and 38.5 years for ritlecitinib. The majority of patients were female (58.5% and 65.0%, respectively). Current AA subtype distribution was comparable between groups (*P* = .710). The majority of patients had a history of severe AA, with alopecia universalis as the worst recorded subtype in 58.5% and 52.5%, respectively. Mean baseline SALT scores were comparable between groups (baricitinib, 81.9 [SD, 26.4]; ritlecitinib, 82.1 [SD, 27.4]; *P* = .967), reflecting severe scalp involvement at treatment initiation. A history of atopy was present in 62.9% and 57.9% of patients. Prior systemic treatment had been received by 89.6% of baricitinib and 95.0% of ritlecitinib patients; no patient in either group received concomitant systemic treatment. Baricitinib was initiated at 4 mg in 96.6% of patients and 2 mg in 3.4% of patients; ritlecitinib at 50 mg in all patients.

**Table I:** Baseline characteristics of patients treated with baricitinib or ritlecitinib

Characteristic	Overall (*n* = 166)	Baricitinib (*n* = 106)	Ritlecitinib (*n* = 60)	*P* value
Age at treatment start, y (mean, SD)	37.8 (14.2)	37.4 (13.9)	38.5 (14.7)	.638
Sex, *n* (%)				
Male	65 (39.2)	44 (41.5)	21 (35.0)	.509
Female	101 (60.8)	62 (58.5)	39 (65.0)	
Age at AA onset, y (mean, SD)	26.5 (15.8)	26.7 (15.9)	26.3 (15.8)	.902
Ethnicity, *n* (%)				
Caucasian	129 (77.7)	81 (76.4)	48 (80.0)	.765
Middle Eastern	23 (13.9)	16 (15.1)	7 (11.7)	
African American	2 (1.2)	2 (1.9)	0 (0)	
Indian	5 (3.0)	2 (1.9)	3 (5.0)	
Hispanic/Latino	3 (1.8)	2 (1.9)	1 (1.7)	
Asian	3 (1.8)	2 (1.9)	1 (1.7)	
Current AA type, *n* (%)				
Universalis	63 (38.0)	42 (39.6)	21 (35.0)	.710
Patchy type	60 (36.1)	39 (36.8)	21 (35.0)	
Totalis	31 (18.7)	17 (16.0)	14 (23.3)	
Ophiasis	12 (7.2)	8 (7.5)	4 (6.7)	
Most severe AA type ever, *n* (%)				
Universalis	93 (56.4)	62 (58.5)	31 (52.5)	.625
Patchy type	33 (20.0)	18 (17.0)	15 (25.4)	
Totalis	29 (17.6)	19 (17.9)	10 (16.9)	
Ophiasis	10 (6.1)	7 (6.6)	3 (5.1)	
SALT score at treatment start (mean, SD)	81.9 (26.7)	81.9 (26.4)	82.1 (27.4)	.967
History of atopy, *n* (%)				
No	63 (38.9)	39 (37.1)	24 (42.1)	.653
Yes	99 (61.1)	66 (62.9)	33 (57.9)	
*Eczema*				
Yes	64 (38.6)	42 (39.6)	22 (36.7)	
*Asthma*				
Yes	32 (19.3)	25 (23.6)	7 (11.7)	
*Allergic rhinitis*				
Yes	72 (43.4)	48 (45.3)	24 (40.0)	
Family history of AA, *n* (%)				
No	126 (77.3)	81 (77.1)	45 (77.6)	1.000
Yes	37 (22.7)	24 (22.9)	13 (22.4)	

Mean (SD) for continuous variables; *n* (%) for categorical variables. *P* values: independent *t* test for continuous variables, χ^2^ or Fisher’s exact test for categorical variables. Atopy subtypes are shown indented under history of atopy and are not mutually exclusive.

*AA*, Alopecia areata; *SALT*, Severity of Alopecia Tool.

### Treatment effectiveness

For the primary LMM, treatment episodes with a baseline SALT score ≤20 were additionally excluded, resulting in 651 observations from 111 baricitinib and 72 ritlecitinib treatment episodes available for multivariable analysis. Both treatments resulted in substantial and sustained reductions in SALT score over time. A mean difference in SALT of Δ37.9 at 9 months and Δ47.3 at 12 months was observed for baricitinib and 34.0 at 9 months and 47.1 at 12 months for ritlecitinib (Supplementary Fig 1, available via Mendeley at https://data.mendeley.com/datasets/x7j3bpv6r7/1).

In the adjusted LMM (Model 2; *n* = 166 patients, 651 observations), SALT score decreased significantly over time at a rate of 4.2 points per month (β = −4.22; 95% CI, −4.64 to −3.79; *P* < .001; Fig 1), confirming the effectiveness of JAKi therapy regardless of treatment choice. No significant difference in SALT score trajectory was observed between baricitinib and ritlecitinib (time × treatment interaction: β = 0.27; 95% CI, −0.49 to 1.03; *P* = .489). The adjusted model was strongly preferred over the unadjusted model based on Akaike information criterion (6012.1 vs 6119.3; difference 107.2 points). Estimated marginal means at 12 months were 33.7 (95% CI, 27.1-40.3) for baricitinib and 33.4 (95% CI, 25.6-41.3) for ritlecitinib (*P* = .956; Table II). The primary finding of no significant difference between baricitinib and ritlecitinib was consistent across all 3 sensitivity analyses. Higher baseline SALT score was the only statistically significant covariate (β = 0.83; 95% CI, 0.65-1.01; *P* < .001), indicating that patients with higher baseline SALT score retained higher SALT scores throughout follow-up. Prior JAKi use showed a trend toward less favorable outcomes (β = 7.74; 95% CI, −0.37 to 15.85; *P* = .061), suggesting that these patients may respond less well to treatment.

**Fig 1:**
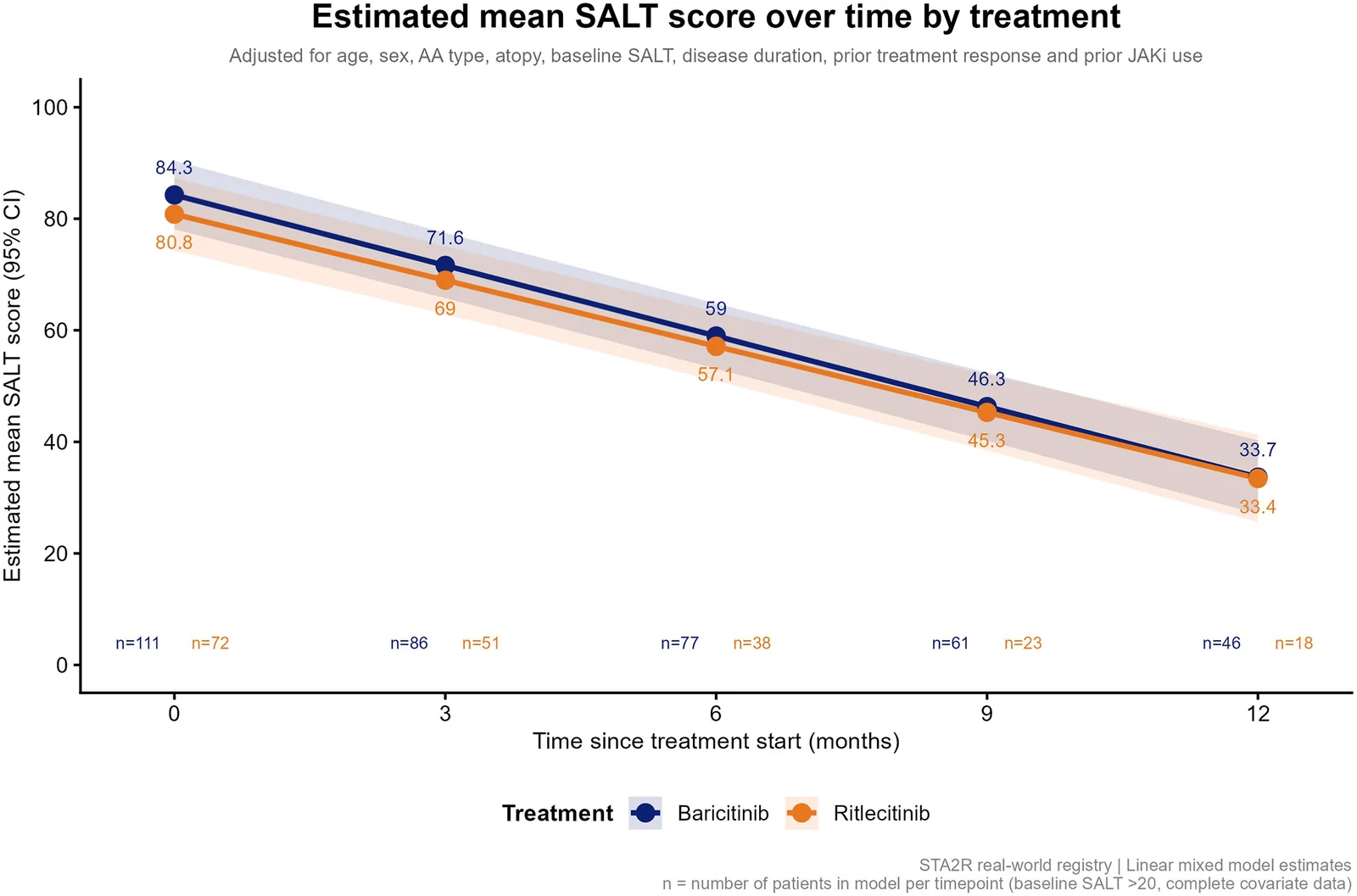
Adjusted Severity of Alopecia Tool (SALT) scores during 12 months of treatment with baricitinib or ritlecitinib. Estimated marginal means with 95% CIs from the adjusted linear mixed model. Lower scores indicate greater scalp hair coverage. *AA*, Alopecia areata; *JAKi*, Janus kinase inhibitor; *STA^2^R*, Systemic Treatments for Alopecia Areata Registry.

**Table II:** Linear mixed model results: SALT score over time (*n* = 651 observations, 166 patients)

Fixed effect	β	SE	df	*t*	*P* value	95% CI
Primary variables						
Time since treatment start (mo)	−**4.22**	0.22	560	−19.34	**<.001**	−4.64 to −3.79
Treatment (ref: baricitinib)						
Ritlecitinib	−3.45	3.25	557	−1.06	.289	−9.84 to 2.93
Time × ritlecitinib interaction	0.27	0.39	576	0.69	.489	−0.49 to 1.03
Covariates						
Age at treatment start (y)	0.05	0.13	173	0.38	.702	−0.20 to 0.30
Sex (ref: male)						
Female	−2.88	3.61	169	−0.80	.426	−10.0 to 4.24
Most severe AA type ever (ref: areata)						
Ophiasis	10.69	7.27	164	1.47	.144	−3.67 to 25.1
Totalis	0.98	6.46	198	0.15	.880	−11.8 to 13.7
Universalis	6.36	5.32	198	1.19	.234	−4.14 to 16.9
History of atopy (ref: no)						
Yes	0.58	3.50	166	0.17	.868	−6.34 to 7.50
SALT score at treatment start	**0.83**	0.09	265	9.06	**<.001**	0.65 to 1.01
Disease duration (years)	0.03	0.16	176	0.18	.856	−0.28 to 0.34
Prior non-JAKi response (ref: treatment-naive)						
No prior response	1.13	5.87	189	0.19	.847	−10.4 to 12.7
Prior response	7.79	5.90	187	1.32	.188	−3.84 to 19.4
Prior JAK inhibitor use (ref: no)						
Yes	7.74	4.10	155	1.89	.061	−0.37 to 15.8

Random intercept per patient included to account for repeated measures.

*AA*, Alopecia areata; *df*, degrees of freedom; *JAKi*, JAK inhibitor; *SALT*, Severity of Alopecia Tool; β, regression coefficient (change in SALT score).

### Response rates (SALT ≤20)

The proportion achieving SALT ≤20 increased progressively over time in both treatment groups. At 9 months, SALT ≤20 was achieved by 31 of 61 (51%) baricitinib patients and 11 of 26 (42%) ritlecitinib patients, increasing to 30 of 47 (64%) and 12 of 24 (50%) at 12 months. No significant difference in time to achieving SALT ≤20 was observed between treatment groups on Kaplan-Meier analysis (log-rank *P* = .65; Supplementary Fig 2, available via Mendeley at https://data.mendeley.com/datasets/x7j3bpv6r7/1).

### Eyebrow, eyelash, body hair, and nail outcomes

At baseline, eyebrow involvement was present in 77 of 106 (73%) and 47 of 60 (78%), eyelash involvement 68 of 106 (64%) and 42 of 60 (70%), body hair involvement 72 of 106 (68%) and 47 of 60 (78%), nail changes 38 of 106 (36%), and 23 of 60 (38%) of baricitinib and ritlecitinib patients, respectively. At 12 months, eyebrow regrowth was observed in 38 of 42 (90%) and 17 of 21 (81%), eyelash regrowth in 33 of 35 (94%) and 12 of 17 (71%), and body hair regrowth in 31 of 39 (79%) and 15 of 21 (71%), for baricitinib and ritlecitinib, respectively. Resolution of nail changes at 12 months was observed in 6 of 13 (46%) of baricitinib and 6 of 9 (67%) of ritlecitinib patients (Supplementary Table I, available via Mendeley at https://data.mendeley.com/datasets/x7j3bpv6r7/1).

### HRQoL

At baseline, HRQoL data were available for 193 treatment episodes from 173 unique patients (baricitinib: 115 episodes, ritlecitinib: 78 episodes). Both groups reported substantial disease burden across all patient-reported outcome measures at baseline, with a median DLQI of 10 (IQR, 5-14) and 7 (IQR, 3.8-12), AASIS of 5.2 (IQR, 3.3-6.5) and 4.4 (IQR, 2.6-5.6), and NRS of 8 (IQR, 7-9) and 7 (IQR, 6-9) for baricitinib and ritlecitinib, respectively. All 3 outcomes (AASIS, DLQI, and NRS) were significantly reduced from baseline at 12 months in both treatment groups, with a mean change of −2.9 points for baricitinib and −2.7 points for ritlecitinib for AASIS (both *P* < .001), −4.8 points for baricitinib (*P* < .001) and −5.1 points for ritlecitinib (*P* = .031) for DLQI, and −3.2 points for baricitinib and −2.5 points for ritlecitinib for NRS (both *P* < .001) (Fig 2).

**Fig 2:**
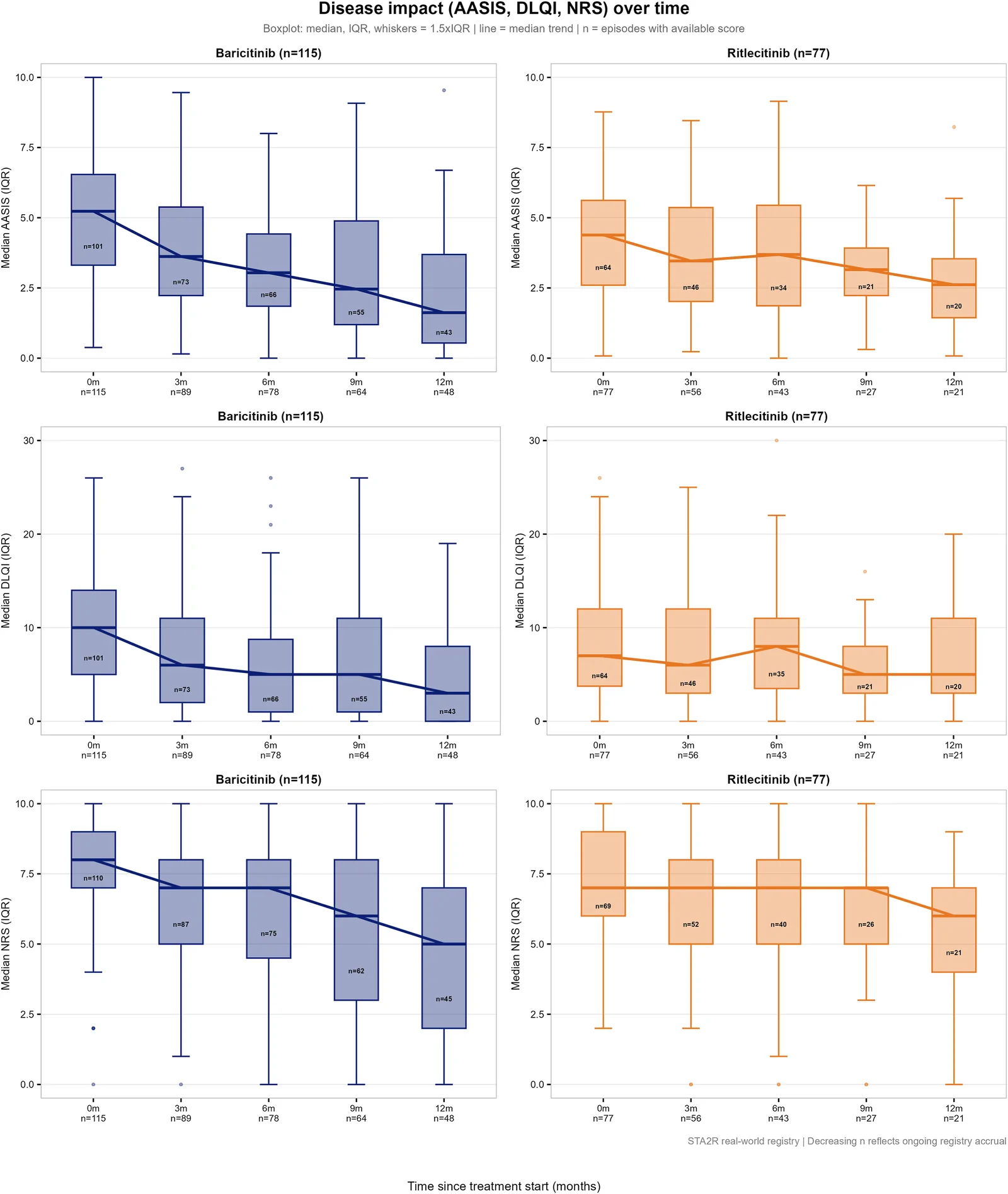
Patient-reported outcomes during treatment with baricitinib or ritlecitinib. Median scores with IQRs for the Alopecia Areata Symptom Impact Scale (AASIS, 0-10), Dermatology Life Quality Index (DLQI, 0-30), and numeric rating scale for disease burden (NRS, 0-10). Lower scores indicate less impact. *STA^2^R*, Systemic Treatments for Alopecia Areata Registry.

### AEs

AEs were reported in 60 (52.2%) baricitinib and 39 (48.1%) ritlecitinib patients (Table III). Infectious complications were most common (28.4%-33.0%), mainly upper respiratory tract infections (25.9%-27.0%). Herpes zoster occurred only in the baricitinib group (2.6%), whereas herpes simplex was more frequent with ritlecitinib (6.2%). Acneiform lesions occurred in 13.0% to 16.0% of patients. Laboratory abnormalities were identified in 6.2% to 13.9%, predominantly elevated liver enzymes (4.9%-5.2%). Treatment was discontinued because of AEs in 2 (1.7%) baricitinib and 3 (3.7%) ritlecitinib patients, because of herpes zoster and hyperlipidemia/weight gain, and urticaria, gastro-intestinal complaints, and suspected ulcerative colitis flare, respectively.

**Table III:** Adverse events by treatment group

Adverse event	Baricitinib (*n* = 115 patients, 117 episodes)		Ritlecitinib (*n* = 81 patients, 81 episodes)	
	Patients, *n* (%)	Events, *n*	Patients, *n* (%)	Events, *n*
Infectious	38 (33.0)	57	23 (28.4)	41
Upper respiratory tract infections	31 (27.0)	39	21 (25.9)	30
Herpes zoster	3 (2.6)	3	0 (0.0)	0
Herpes simplex	1 (0.9)	1	5 (6.2)	7
Urinary tract infection	0 (0.0)	0	2 (2.5)	3
Local skin infections∗	7 (6.1)	10	0 (0.0)	0
Other infections†	3 (2.6)	4	1 (1.2)	1
Gastro-intestinal	9 (7.8)	13	11 (13.6)	15
Neurological	18 (15.7)	26	2 (2.5)	2
Laboratory abnormalities	16 (13.9)	26	5 (6.2)	8
Elevated liver enzymes	6 (5.2)	8	4 (4.9)	4
Hyperlipidemia	5 (4.3)	6	1 (1.2)	1
Decreased eGFR	4 (3.5)	5	1 (1.2)	1
Hematologic abnormalities‡	4 (3.5)	7	0 (0.0)	0
Other laboratory abnormalities§	0 (0.0)	0	1 (1.2)	2
Dermatological	20 (17.4)	34	18 (22.2)	23
Acne	15 (13.0)	26	13 (16.0)	18
Urticaria	0 (0.0)	0	2 (2.5)	2
AD flare	1 (0.9)	2	1 (1.2)	1
Other||	5 (4.3)	6	2 (2.5)	2
General	11 (9.6)	17	7 (8.6)	7
Fatigue	8 (7.0)	10	3 (3.7)	3
Weight gain	2 (1.7)	2	3 (3.7)	3
Other¶	4 (3.5)	5	1 (1.2)	1
Other#	7 (6.1)	7	7 (8.6)	7

*AD*, Atopic dermatitis; *eGFR*, estimated glomerular filtration rate.

∗ Local skin infections include: folliculitis, impetigo (ear), paronychia, panaritium, periorbital cellulitis, tinea corporis, and skin infection (neck).

† Other infections includes: ear infection, sinusitis, and tooth infection.

‡ Hematologic abnormalities includes: thrombocytosis and decreased hemoglobin.

§ Other laboratory abnormalities includes: creatine kinase elevation (2× upper limit of normal).

|| Other (dermatological) includes: xerosis cutis, seborrhoeic eczema (scalp), chalazion, rosacea, erythema (mouth), and rash face.

¶ Other (general) includes: flushing, edema/fluid retention, chest tightness, atypical chest pain, dry eyes, muscle pain, burning sensation in hands/feet, and increase in asthma symptoms.

# Other includes: aphthous ulcers, decreased appetite, increased appetite, decreased libido, changed menstrual pattern, increased endometriosis complaints, flank pain, nail pain, ecchymosis, lymphadenopathy (reactive), increased fibromyalgia complaints, and suspected ulcerative colitis flare.

## Discussion

To our knowledge, this registry (STA^2^R) provides the first prospective, real-world data on the systemic treatment of AA in the Netherlands. In this cohort of adults with predominantly severe disease, treatment with baricitinib or ritlecitinib resulted in substantial and sustained improvement in scalp hair regrowth. Mean SALT scores declined by approximately 60% over 12 months, and 30 of 47 (64%) and 12 of 24 (50%) of baricitinib and ritlecitinib patients, respectively, achieved at least 80% scalp hair coverage (SALT ≤20) by 12 months. These findings confirm the real-world effectiveness of JAKi and support their role as effective treatments for severe AA.

The main finding of this study is that baricitinib and ritlecitinib demonstrated similar effectiveness in terms of scalp hair regrowth. No significant difference in SALT score trajectory was observed between treatment groups after multivariable adjustment (time × treatment interaction: β = 0.27, *P* = .489), and estimated marginal means at 12 months were almost identical. This finding was consistent across all 3 sensitivity analyses, including complete exclusion of within-registry switchers, confirming its robustness.

The magnitude of improvement in STA^2^R appears to be higher than that reported in randomized controlled trials, such as BRAVE-AA1/2 and ALLEGRO, where 35% to 47% of patients achieved similar end points.^6^, ^7^, ^8^, ^9^ This difference may reflect several factors inherent to real-world practice: less restrictive inclusion and exclusion criteria compared with clinical trials, longer treatment duration in responding patients, greater treatment flexibility, fewer protocol-mandated withdrawals, and reduced dropout of potential responders. Importantly, similar findings have been reported in other real-world or retrospective cohorts,^10^, ^11^, ^13^ suggesting that the clinical benefit of JAKi may even be more pronounced outside controlled trial settings.

Response rates continued to increase up to 12 months in both groups, indicating that regrowth accumulates with prolonged treatment. These findings reinforce that severe AA behaves as a chronic condition requiring long-term immunomodulation rather than short-term therapy. Beyond scalp hair, both treatments resulted in meaningful improvements in eyebrow, eyelash, and body hair regrowth, with regrowth observed in the large majority of patients with involvement at baseline by 12 months. An intriguing finding was the difference in nail change resolution between treatment groups, with resolution overall appearing to lag behind other AA-associated features. Although this observation warrants further investigation, it should be interpreted with caution given the small sample sizes, binary assessment without a validated scoring instrument, and the descriptive nature of this analysis.

Our results add to the growing body of evidence supporting the effectiveness of JAKi in diverse clinical settings.^19^ The overall response rate in STA^2^R aligns with recent real-world studies from Italy and the Netherlands.^10^, ^11^ These studies, together with ours, suggest that JAK inhibition provides durable regrowth across ethnic backgrounds and treatment histories.^20^ The similar performance of baricitinib and ritlecitinib in this registry supports the idea that both JAK1/2 and JAK3/tyrosine kinase expressed in hepatocellular carcinoma family kinase inhibition are viable strategies for targeting the shared inflammatory cascade in AA. Longer-term follow-up will be needed to determine whether these mechanisms differ in durability of response, relapse patterns, or AEs.

This study has several limitations. As an observational registry, it lacks randomization and a control group, which may introduce confounding by indication despite multivariable adjustment. Additionally, the unequal, nonpowered treatment groups mean that this was not a head-to-head comparison, and between-group differences should be interpreted descriptively. Decreasing sample sizes at later time points largely reflect ongoing accrual and active follow-up rather than systematic dropout, but limit precision at later time points. Nail and facial/body hair regrowth outcomes were assessed using a binary classification without a validated scoring instrument, limiting quantitative interpretation. The single-country, predominantly Caucasian cohort limits external validity to other populations or health care settings.

Despite these limitations, the prospective design, standardized SALT assessment at each visit, and multicenter participation strengthen the validity and generalizability of these findings. The STA^2^R registry complements trial data by providing real-world evidence on the effectiveness of JAKi under routine clinical conditions. Future analyses will explore long-term safety, JAKi switchers, predictors of treatment durability and relapse, and optimal treatment duration. By integrating real-world evidence with existing trial data, STA^2^R may ultimately help guide personalized treatment decisions in the evolving management of AA.

## Conclusion

In this prospective real-world registry, baricitinib and ritlecitinib demonstrated similar scalp hair regrowth, with responses continuing to improve up to 12 months and consistent improvements in HRQoL. These findings support both JAKi as effective, well-tolerated real-world treatments for AA.

### Declaration of generative AI and AI-assisted technologies in the writing process

During the preparation of this work, the authors used Claude (Anthropic) in order to support language editing and to improve the readability of the manuscript. After using this tool, the authors reviewed and edited the content as needed and take full responsibility for the content of the published article.

## Conflicts of interest

Dr Hijnen has served as principal investigator on clinical trials in atopic dermatitis for Sanofi, AbbVie, Galderma, and Priovant; has conducted investigator-initiated studies supported by UCB, AbbVie, and LEO Pharma; and has served on advisory boards for UCB, Sanofi, and Almirall; all compensation, honoraria, and payments related to these activities were made to his institution; he has no personal financial interests. Dr Waalboer-Spuij has served on an advisory board for AbbVie. Drs Willaert, Helmond, Vugt, Gostyński, Dongen, Janmohamed, Zweers, and Nijsten no conflicts of interest to declare.
